# Learning curve for open surgical repair of acute type A aortic dissection

**DOI:** 10.1038/s41598-023-30397-2

**Published:** 2023-03-03

**Authors:** Bo-Cheng Hou, Yu-Tung Huang, Fu-Chih Hsiao, Chien-Chia Wu, Yu-Ting Cheng, Kuo-Sheng Liu, Shang-Hung Chang, Pao-Hsien Chu, An-Hsun Chou, Shao-Wei Chen

**Affiliations:** 1 Chiayi Branch, Chang Gung Memorial Hospital, No. 8, Sec. W., Jiapu Rd., Puzi City, Chiayi County Taiwan; 2 Division of Thoracic and Cardiovascular Surgery, Department of Surgery, Linkou Medical Center, Chang Gung Memorial Hospital, Chang Gung University, No. 5 Fuxing Street, Guishan District, Taoyuan City, 33305 Taiwan; 3 Center for Big Data Analytics and Statistics, Linkou Medical Center, Chang Gung Memorial Hospital, Taoyuan City, Taiwan; 4 Department of Cardiology, Linkou Medical Center, Chang Gung Memorial Hospital, Chang Gung University, Taoyuan City, Taiwan; 5 Department of Anesthesiology, Linkou Medical Center, Chang Gung Memorial Hospital, Chang Gung University, Taoyuan City, Taiwan; 6 Linkou Medical Center, Chang Gung Memorial Hospital, Chang Gung University, Taoyuan City, Taiwan

**Keywords:** Cardiovascular biology, Interventional cardiology, Cardiology

## Abstract

There is scarce evidence about the surgeon learning curve of acute type A aortic dissection surgery and whether the optimal procedure number exists when training a cardiovascular surgeon. A total of 704 patients with acute type A aortic dissection surgery performed by 17 junior surgeons who can identify their first career surgery from January 1, 2005, to December 31, 2018, are included. The surgeon experience volume is defined as the cumulative number of acute type A aortic dissection surgery of the surgeon since January 1, 2005. The primary outcome was in-hospital mortality. The possibility of non-linearity and cutoffs for surgeon experience volume level was explored using a restricted cubic spline model. The results revealed that more surgeon experience volume is significantly correlated to a lower in-hospital mortality rate (*r* = − 0.58, *P* = 0.010)*.* The RCS model shows for an operator who reaches 25 cumulative volumes of acute type A aortic dissection surgery, the average in-hospital mortality rate of the patients can be below 10%. Furthermore, the longer duration from the 1st to 25th operations of the surgeon is significantly correlated to a higher average in-hospital mortality rate of the patients (*r* = 0.61, *p* = 0.045). Acute type A aortic dissection surgery has a prominent learning curve in terms of improving clinical outcomes. The findings suggest fostering high-volume surgeons at high-volume hospitals can achieve optimal clinical outcomes.

## Introduction

Acute type A aortic dissection (ATAAD), the most lethal disease among acute aortic syndromes^1^, is an emergent condition and one of the most fatal diseases requiring immediate diagnosis and surgical intervention^2^. Although ATAAD is rare—with an incidence of 4 to 19 per 100 000 person-years^3–5^—its high mortality and rapid progression highlight the urgency for surgical intervention^6,7^. Surgical intervention has always been the optimal treatment for ATAAD^8^. A report based on the international registry of acute aortic dissection indicated that an increase in the trend of surgical management of ATAAD was correlated with a decrease in the mortality rate from 25% in the late 1990s to 18% in the 2010s^9^; this decrease is attributable to early detection, improved diagnostic methods, and advances in surgical techniques and postoperative care.

Despite advancements in knowledge and techniques regarding ATAAD treatment, in-hospital mortality has been prevalent, according to records from different populations and database^3,4,10,11^; this signifies the lethality of ATAAD pathogenesis and the need for a treatment technique with high proficiency and reliability^12^. ATAAD is an emergent disease with unpredictable characteristics. Accordingly, most patients with ATAAD may arrive at the emergency department unexpectedly and be operated on by an on-duty surgeon rather than an elective experienced surgeon^13^. Hence, a surgeon’s proficiency and adaptability play crucial roles in such a challenging situation.

Several studies have explored the hospital volume–outcome relationship and surgeon total volume–outcome relationship for ATAAD surgical management^11,14–16^ and have demonstrated favorable outcomes with increased volume. However, scarce evidence is available regarding the surgeon learning curve for surgical repair of ATAAD and the optimal operative volume (number of surgical procedures) exists for a cardiovascular surgeon under training. We hypothesized that accumulating surgical experience would be associated with improved surgical outcomes for ATAAD and that a precise operative volume exists for a surgeon to achieve consistently favorable surgical results. Accordingly, we retrospectively analyzed surgeons’ sequential performance of ATAAD surgery and explored the optimal number of procedures (operative volume) required for a junior surgeon to achieve ideal surgical outcomes.

## Methods

### Data source

Patient data were retrieved from the Chang Gung Research Database (CGRD). The CGRD includes detailed original medical records, such as laboratory reports, hemodynamic records, physical examination records, and medical imaging reports, from the Chang Gung Memorial Hospital system, which is composed of 3 tertiary medical centers and 4 regional medical institutions located from the northeast to southeast regions of Taiwan. Moreover, the CGRD covers 21.2% and 12.4% of the total national outpatient and inpatient visits, respectively^17^; it includes data on > 4 million outpatient visits, 200 000 emergency department visits, and 1 000 000 inpatient visits annually. This study was approved by the Institutional Review Board of Chang Gung Memorial Hospital (IRB No.: 202100124B0), and all methods in the article were performed in accordance with the guidelines and regulations of it. The need for informed consent was waived by the Institutional Review Board of Chang Gung Memorial Hospital.

### Study population

**Figure 1:**
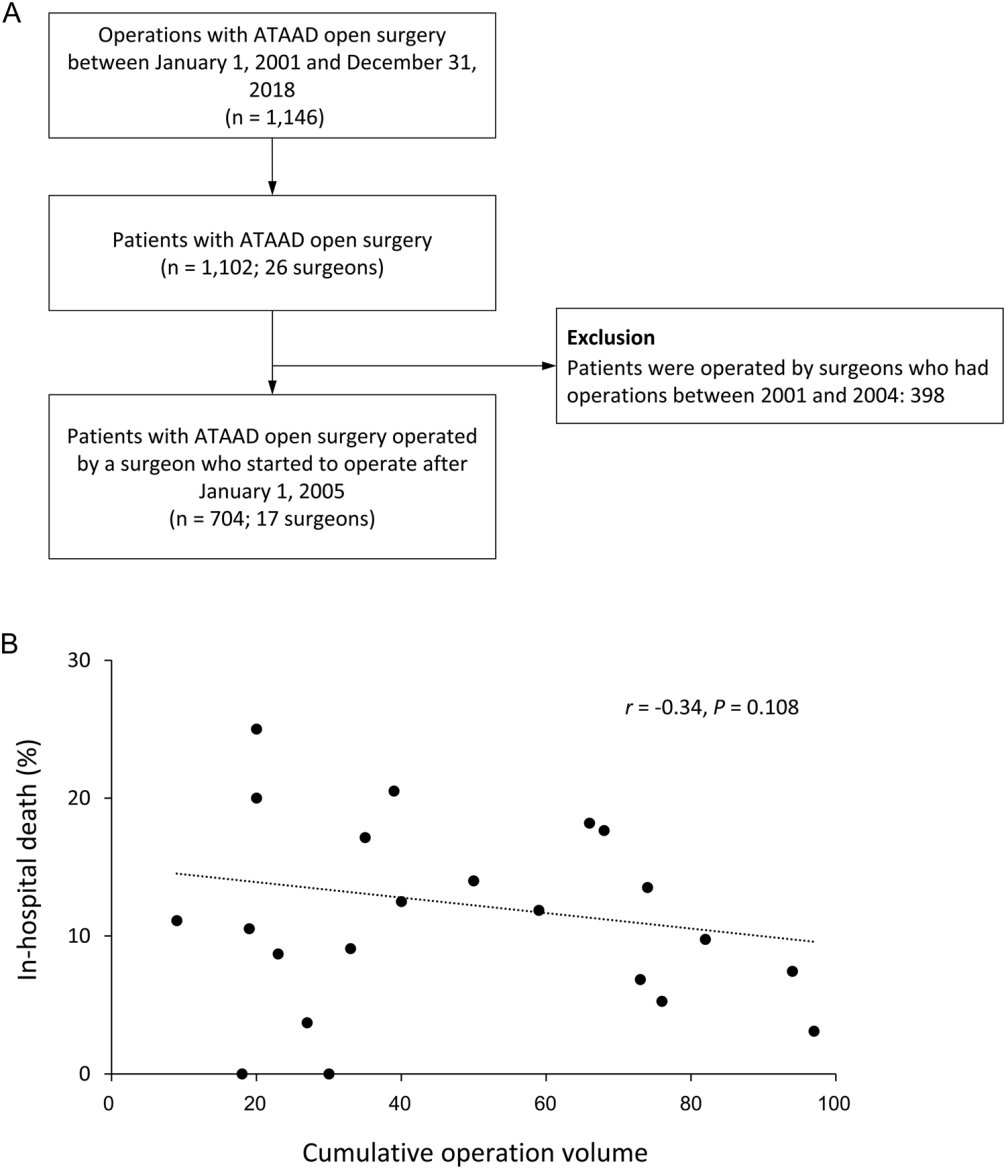
The flowchart for inclusion and exclusion of the study patients (**A**) and the relationship between cumulative operation volume of the surgeon (from 2001 to 2018) and the average in-hospital mortality rate of patients who are operated by the surgeon (**B**). Each point consists of all patients among the operations of the surgeon. Only those surgeons who conducted more than 10 operations are included in the plot. ATAAD, acute type A aortic dissection.

Since the electronic records of CGRD initiated in 2001, patients with ATAAD who underwent open surgery due to an acute onset of aortic dissection between January 1, 2001, and December 31, 2018, were consecutively included. The presence of ATAAD and corresponding open surgery was ascertained through the examination of discharge diagnoses, operation notes, and discharge notes recorded in the CGRD subsets. These records were confirmed by 2 experienced cardiovascular surgeons. To evaluate the effect of the learning curve on perioperative outcomes, patients who were operated on by the surgeons (either senior or junior) who had surgical records between 2001 and 2004 were excluded from the main analysis (Fig. 1A). In other words, we only included patients whose surgeons started to operate since January 1, 2005. It’s very unlikely that one surgeon conducted surgical repair of ATAAD before 2001 but did not conduct between 2001 and 2004. Therefore, to identify a surgeon’s first career surgical repair of ATAAD, the learning curve was defined as the sequential number of open surgical procedures for ATAAD performed by the surgeon after January 1, 2005.

### Outcomes

The primary outcome was in-hospital mortality, defined as death due to any reason during the index admission. The secondary outcomes comprised composite events (in-hospital mortality, new-onset stroke, new-onset hemodialysis, respiratory failure, use of extracorporeal membrane oxygenation, or massive blood transfusion [defined as packed red blood cell > 10 U]) during the index admission, bypass time, clamp time, and arrest time in surgery; length of intensive care unit (ICU) stay; and length of hospital stay. Information regarding the aforementioned outcomes was extracted from the death registry, inpatient claims data, operation notes, and discharge notes in the CGRD subsets.

### Covariates

Covariates were age, sex, body weight, body height, body mass index, presenting complications (e.g. pain with radiation or chest pain or tightness), comorbid conditions (e.g. hypertension, coronary heart disease, end-stage renal disease, previous cardiac surgery history, or Marfan syndrome), severity of ATAAD (e.g. intramural hematoma, deBakey I, deBakey II), preoperative malperfusion syndrome, preoperative tamponade or shock, preoperative laboratory data (e.g. creatinine levels or whole blood cell count), postoperative laboratory data (e.g. platelet or hemoglobin levels), brain protection type, histidine-tryptophan-ketoglutarate solution volume, cerebral perfusion volume, and surgical extension for ATAAD (partial or total aortic arch replacement, aortic root replacement, elephant trunk, and ascending aorta replacement only). Information regarding the aforementioned covariates was extracted from the outpatient and inpatient claims data, laboratory records, computed tomography reports, operation notes, and discharge notes of the CGRD subsets.

### Statistical analysis

First, surgeon operative volume is presented as a categorical variable stratified into 4 groups according to the sequential number of surgical procedures performed by surgeons: group 1 (comprising surgeries performed by surgeons’ 1st to 10th volume), group 2 (comprising surgeries performed by surgeons’ 11th to 20th volume), group 3 (comprising surgeries performed by surgeons’ 21st to 30th volume), and group 4 (comprising surgeries performed by surgeons’ > 30th volume). The linear trend of outcome across these groups was assessed using linear contrast analysis in a general linear model for continuous outcomes (i.e. clamp time) and the Cochran-Armitage trend test for categorical outcomes (i.e. in-hospital death). Due to the lack of normality, the linear trends of length of ICU stay and length of hospital stay across the ordinal groups of operative volume were evaluated using the nonparametric Jonckheere-Terpstra test.

Second, the relationship between surgeon operative volume (continuous variable) and the mean value of an outcome was assessed using Pearson’s correlation. Only a few surgeons (n = 13) had performed > 20 operations at the time of the study. Therefore, surgeon operative volume was further stratified based on different strategies according to the sequential number of operations performed on patients: the first stratification comprised surgeons performing their 1st to 19th operations (the sequential numbers of operative volumes per dot: 2), second stratification comprised those performing their 20th to 39th operations (the sequential numbers of operative volumes per dot: 5), and third stratification comprised those performing their > 40th operations (the sequential numbers of operative volumes per dot: 10).

Since the choice of the cutoffs of the surgical volume above mentioned is arbitrary, we conducted an alternative model, of which the surgeon operative volume was treated as a restricted cubic spline (RCS) variable in the logistic regression model on in-hospital mortality. The possibility of nonlinearity and the potential cutoffs for optimal surgeon operative volume were explored in the RCS model. The number of knots was 4, and the locations were 5th, 35th, 65th, and 95th percentiles. Finally, the relationship between the duration from the 1st to 25th operations of the surgeon and in-hospital mortality rate was assessed using Pearson’s correlation. RCS modeling was performed using R (version 4.0.2; R Foundation for Statistical Computing) and the RMS package (version 5.1–3.1; Frank E. Harrell Jr). Other statistical analyses were performed using SAS (version 9.4; SAS Institute, Cary, NC, USA). A 2-sided *P* value of < 0.05 was considered statistically significant.

## Result

Of the 1102 patients with ATAAD who received open surgery (performed by 26 surgeons) between 2001 and 2018, 398 were excluded because they received surgery from senior surgeons (defined as surgeons who performed their first operation before January 1, 2005); we removed these patients to purify and clarify the learning curve for surgical repair of ATAAD, by precisely identifying a surgeon’s first career ATAAD surgery. The remaining 704 patients (who received surgery from 17 junior surgeons) were selected in the analysis of surgeons’ sequential performance of surgical repair of ATAAD (Fig. 1A).

For the 26 surgeons who were initially included from 2001 to 2018, the relationship between their total cumulative operative volumes and average in-hospital mortality rate of the patients who received their operations is illustrated in Fig. 1B. Only surgeons who had conducted > 10 operations were included in this analysis. The results revealed that a high cumulative operative volume was modestly correlated with a low in-hospital mortality rate (correlation coefficient [*r*] =  − 0.34), although the correlation was not significant (*P* = 0.108). Furthermore, for the 17 remaining junior surgeons included from 2005 to 2018, detailed information regarding the cumulative operative volumes, in-hospital mortality, and composite events for patients is presented in Supplemental Table 1.

Among the remaining 704 patients, 153 were operated on by surgeons in group 1 (i.e. surgeons performing their 1st to 10th operations), 130 were operated on by those in group 2 (surgeons performing their 11th to 20th operations), 110 were operated on by those in group 3 (surgeons performing their 21st to 29th operations), and 331 were operated on by those in group 4 (surgeons performing their > 31st operations). The mean age and the proportion of male patients were 58 years and approximately 67% among all groups, respectively. The mean duration between the arrival time in the emergency department and the time entering the operation room was 1.8 h in average. For malperfusion syndrome, 35.5% patients had any aortic vascular branch involved, which did not demonstrate significant difference among groups. The majority of the patients presented with a chief concern of chest pain or back pain; 70.3% of the patients had a history of hypertension, 2.4% had Marfan syndrome, 1.7% had end stage renal disease receiving dialysis, and 1.56% received cardiac surgery previously. Regarding surgical procedures, 59.2% of the patients received surgery confined to the ascending aorta only, and 31.8, 6.7 and 3.3% of the patients received surgery that extended to the aortic arch, involved the Bentall procedure, and involved the frozen elephant trunk procedure, respectively. The differences in comorbidities and preoperative laboratory data across the 4 groups were nonsignificant (Table 1).

**Table 1:** Baseline characteristics of the patients according to the cumulative operation volume of the surgeon.

Variable	Valid *N*	Total (*N* = 704)	Number of ATAAD surgery of the surgeon			
			≤ 10th (n = 153)	11th to 20th (n = 130)	21th to 30th (n = 110)	> 30th (n = 311)
Demographics						
Age, year	704	58.4 ± 13.7	58.8 ± 12.9	57.2 ± 13.7	57.5 ± 14.0	59.1 ± 14.1
Male	704	473 (67.2)	103 (67.3)	92 (70.8)	75 (68.2)	203 (65.3)
Body weight, kg	704	73.1 ± 16.3	73.5 ± 16.0	75.2 ± 18.7	70.3 ± 14.0	72.9 ± 16.0
Body height, cm	704	164.7 ± 14.2	163.9 ± 14.8	165.8 ± 15.0	164.3 ± 15.5	164.7 ± 13.1
BMI, kg/m^2^	704	29.0 ± 30.0	28.7 ± 15.9	31.6 ± 49.8	30.2 ± 44.2	27.6 ± 14.0
Presenting complications						
Pain with radiation	704	77 (10.9)	23 (15.0)	13 (10.0)	14 (12.7)	27 (8.7)
Chest pain or tightness	704	387 (55.0)	73 (47.7)	75 (57.7)	71 (64.6)	168 (54.0)
Back pain	704	142 (20.2)	41 (26.8)	25 (19.2)	18 (16.4)	58 (18.7)
Abdominal pain	704	50 (7.1)	12 (7.8)	7 (5.4)	8 (7.3)	23 (7.4)
Neck or head pain	704	14 (2.0)	4 (2.6)	1 (0.8)	4 (3.6)	5 (1.6)
Peripheral limbs weakness or numbness	704	42 (6.0)	11 (7.2)	7 (5.4)	2 (1.8)	22 (7.1)
Dyspnea	704	22 (3.1)	6 (3.9)	5 (3.9)	5 (4.6)	6 (1.9)
Syncope	704	72 (10.2)	15 (9.8)	20 (15.4)	7 (6.4)	30 (9.7)
Others	704	56 (8.0)	9 (5.9)	7 (5.4)	8 (7.3)	32 (10.3)
Duration between the onset (the arrival at the emergent department) and entering the operating room, hrs	690	1.8 [1.2—3.35]	2.5 [1.5—4.9]	2 [1.3—3.4]	1.9 [1.2—2.9]	1.6 [1—2.8]
Malperfusion						
Coronary	704	21 (3.0)	3 (2.0)	3 (2.3)	4 (3.6)	11 (3.5)
Left cerebral	704	32 (4.5)	7 (4.6)	6 (4.6)	0 (0.0)	19 (6.1)
Right cerebral	704	64 (9.1)	14 (9.2)	11 (8.5)	11 (10.0)	28 (9.0)
Celiac trunk	704	39 (5.5)	10 (6.5)	2 (1.5)	7 (6.4)	20 (6.4)
Mesenteric	704	37 (5.3)	13 (8.5)	5 (3.9)	3 (2.7)	16 (5.1)
Left renal	704	80 (11.4)	24 (15.7)	10 (7.7)	15 (13.6)	31 (10.0)
Right renal	704	65 (9.2)	16 (10.5)	12 (9.2)	13 (11.8)	24 (7.7)
Left lower limb	704	43 (6.1)	8 (5.2)	8 (6.2)	6 (5.5)	21 (6.8)
Right lower limb	704	47 (6.7)	9 (5.9)	7 (5.4)	5 (4.6)	26 (8.4)
Any of above	704	250 (35.5)	59 (38.6)	43 (33.1)	38 (34.6)	110 (35.4)
Severity	702					
IMH		133 (18.9)	26 (17.0)	20 (15.4)	23 (20.9)	64 (20.7)
deBakey I		562 (80.1)	127 (83.0)	109 (83.9)	86 (78.2)	240 (77.7)
deBakey II		7 (1.0)	0 (0.0)	1 (0.8)	1 (0.9)	5 (1.6)
Aortic valve involvement (aortic valve replacement or Bentall root surgery)	704	58 (8.2)	6 (3.9)	10 (7.7)	11 (10.0)	31 (10.0)
Comorbidities						
Hypertension	704	495 (70.3)	112 (73.2)	92 (70.8)	74 (67.3)	217 (69.8)
Coronary heart disease	704	58 (8.2)	18 (11.8)	11 (8.5)	10 (9.1)	19 (6.1)
Marfan syndrome	704	17 (2.4)	2 (1.3)	5 (3.9)	3 (2.7)	7 (2.3)
Previous cardiac surgery	704	11 (1.6)	0 (0.0)	2 (1.5)	1 (0.9)	8 (2.6)
Diabetes mellitus	704	53 (7.5)	8 (5.2)	10 (7.7)	4 (3.6)	31 (10.0)
Chronic kidney disease	704	104 (14.8)	24 (15.7)	15 (11.5)	15 (13.6)	50 (16.1)
Dialysis	704	12 (1.7)	2 (1.3)	2 (1.5)	2 (1.8)	6 (1.9)
Liver disease	704	77 (10.9)	19 (12.4)	13 (10.0)	15 (13.6)	30 (9.7)
Atrial fibrillation	704	49 (7.0)	14 (9.2)	8 (6.2)	6 (5.5)	21 (6.8)
COPD	704	31 (4.4)	6 (3.9)	6 (4.6)	6 (5.5)	13 (4.2)
Old stroke	704	28 (4.0)	7 (4.6)	7 (5.4)	2 (1.8)	12 (3.9)
Previous cardiac surgery	704	11 (1.6)	0 (0.0)	2 (1.5)	1 (0.9)	8 (2.6)
Pre-op conditions						
Tamponade/Shock	704	106 (15.1)	25 (16.3)	24 (18.5)	5 (4.6)	52 (16.7)
Preoperative lab data						
Creatinine, mg/dL	681	1.4 ± 1.4	1.5 ± 1.7	1.3 ± 0.9	1.4 ± 0.9	1.5 ± 1.6
WBC, 10^3^/uL	699	13.1 ± 4.8	12.8 ± 4.3	13.1 ± 4.6	12.5 ± 4.5	13.4 ± 5.2
Platelet, 1000/uL	699	179.6 ± 66.5	176.7 ± 64.8	173.6 ± 55.2	181.0 ± 60.3	183.1 ± 73.5
Hemoglobin, g/dL	699	13.4 ± 2.1	13.5 ± 2.0	13.5 ± 2.1	13.5 ± 1.9	13.2 ± 2.1
BUN, mg/dL	499	19.7 ± 11.6	21.2 ± 12.0	19.7 ± 10.4	19.5 ± 11.1	19.1 ± 12.2
Sodium, mg/dL	691	138.9 ± 3.4	139.0 ± 3.8	139.3 ± 3.8	139.2 ± 2.8	138.6 ± 3.3
Potassium, mg/dL	692	3.8 ± 0.6	3.7 ± 0.5	3.8 ± 0.5	3.8 ± 0.6	3.8 ± 0.6
Albumin, mg/dL	171	3.2 ± 0.6	3.3 ± 0.5	3.4 ± 0.5	3.2 ± 0.6	3.2 ± 0.6
HbA1c, %	181	6.0 ± 0.7	6.0 ± 0.7	6.0 ± 0.6	5.9 ± 0.4	6.1 ± 0.8
AST, U/L	413	38 [26, 64]	38 [26, 63]	42 [30, 72]	37 [25, 67]	37 [26, 65]
ALT, U/L	493	26 [19, 43]	24 [19, 38]	29 [19, 53]	23 [17, 44]	26 [19, 43]
INR	677	1.15 ± 0.25	1.16 ± 0.37	1.14 ± 0.16	1.12 ± 0.13	1.15 ± 0.24
Post-operative lab data						
Platelet, 1000/uL	672	136.4 ± 45.0	131.5 ± 42.8	137.2 ± 44.2	147.8 ± 52.0	134.5 ± 43.2
Hemoglobin, g/dL	672	10.7 ± 1.7	10.9 ± 1.6	10.9 ± 1.7	10.8 ± 1.7	10.6 ± 1.6
AST, U/L	469	85 [54, 174]	91 [58, 177]	102 [57, 187]	76 [57, 141]	81 [53, 189]
ALT, U/L	458	45 [25, 101]	43 [23, 103]	55 [27, 111]	46 [27, 93]	44 [25, 105]
Lactic acid	253	62.9 ± 47.9	71.3 ± 40.2	81.0 ± 76.8	53.4 ± 32.2	59.7 ± 43.4
Proteinuria	183	30 [15, 100]	30 [15, 100]	30 [15, 100]	30 [0, 100]	30 [15, 100]
SOFA score	276	10.7 ± 2.2	11.1 ± 1.9	10.4 ± 1.9	10.7 ± 2.4	10.7 ± 2.3
Surgical data						
Brain protection	704					
Antegrade		368 (52.3)	62 (40.5)	65 (50.0)	59 (53.6)	182 (58.5)
Retrograde		336 (47.7)	91 (59.5)	65 (50.0)	51 (46.4)	129 (41.5)
HTK, cc	303	2196 ± 573	2003 ± 553	2225 ± 561	2134 ± 530	2250 ± 588
Cerebral perfusion, min	298	49.0 ± 22.0	44.7 ± 17.9	50.0 ± 23.0	44.5 ± 14.5	51.6 ± 23.9
Surgical extension						
Partial or total aortic arch replacement	704	224 (31.8)	56 (36.6)	43 (33.1)	41 (37.3)	84 (27.0)
Aortic root replacement	704	47 (6.7)	4 (2.6)	6 (4.6)	10 (9.1)	27 (8.7)
Elephant trunk	704	23 (3.3)	1 (0.7)	5 (3.9)	1 (0.9)	16 (5.1)
Ascending aorta replacement only	704	417 (59.2)	93 (60.8)	77 (59.2)	58 (52.7)	189 (60.8)

*ATAAD* acute type A aortic dissection, *BMI* body mass index, *IMH* intramural hematoma, *COPD* chronic obstructive pulmonary disease, *WBC* whole blood cell, *BNP* B-type natriuretic peptide, *BUN* blood urea nitrogen, *HCO*_*3*_ bicarbonate, *HbA1c* glycated hemoglobin, *AST* aspartate aminotransferase, *ALT* alanine aminotransferase, *INR* international normalized ratio.

Data were presented as frequency (percentage) or mean ± standard deviation or median [Quartile 1, Quartile 3].

We treated surgeon operative volume as a categorical variable (Table 2). The results indicated that the in-hospital mortality rate (*P* for trend = 0.031) exhibited a significant decreasing trend as the surgeon operative volume increased. We conducted the subgroup analysis by several variables, including any surgical extension, malperfusion syndrome, tamponade/shock, duration between the onset and entering the operating room, extent of dissection / severity, and aortic valve involvement (Table 2). The effect of surgeon operative volume was more pronounced in patients with any surgical extension (*P* for trend = 0.080), without malperfusion syndrome (*P* for trend = 0.056), with tamponade/shock (*P* for trend = 0.022), with longer duration between the onset and entering the operating room (*P* for trend < 0.001), with deBakey I (*P* for trend = 0.032) and without aortic valve involvement (*P* for trend = 0.022).

**Table 2:** In-hospital mortality of the patients according to the cumulative operation volume of the surgeon by different subgroups.

Subgroup	No. of patients	Total	Number of ATAAD surgery of the surgeon				*P* trend
			≤ 10th	11th to 20th	21st to 30th	> 30th	
Overall		76 (10.8)	23 (15.0)	17 (13.1)	8 (7.3)	28 (9.0)	0.031
Surgical extension							
No (ascending only)	417	37 (8.9)	12 (12.9)	7 (9.1)	3 (5.2)	15 (7.9)	0.184
Yes (any extension)	287	39 (13.6)	11 (18.3)	10 (18.9)	5 (9.6)	13 (10.7)	0.080
Malperfusion syndrome							
No	454	42 (9.3)	11 (11.7)	13 (14.9)	4 (5.6)	14 (7.0)	0.056
Yes	250	34 (13.6)	12 (20.3)	4 (9.3)	4 (10.5)	14 (12.7)	0.290
Tamponade/Shock							
No	598	58 (9.7)	16 (12.5)	12 (11.3)	6 (5.7)	24 (9.3)	0.249
Yes	106	18 (17.0)	7 (28.0)	5 (20.8)	2 (40.0)	4 (7.7)	0.022
Duration between the onset (the arrival at the emergent department) and entering the operating room							
< 1.8 h	323	37 (11.5)	5 (9.1)	7 (13.2)	5 (10.2)	20 (12.1)	0.691
≥ 1.8 h	365	38 (10.4)	18 (18.8)	10 (13.5)	3 (5.6)	7 (5.0)	< 0.001
Extent of dissection/Severity							
IMH	133	8 (6.0)	2 (7.7)	1 (5.0)	0 (0.0)	5 (7.8)	0.883
deBakey I	562	68 (12.1)	21 (16.5)	16 (14.7)	8 (9.3)	23 (9.6)	0.032
deBakey II	7	0 (0.0)	NA	0 (0.0)	0 (0.0)	0 (0.0)	NA
Aortic valve involvement							
No	646	71 (11.0)	22 (15.0)	17 (14.2)	8 (8.1)	24 (8.6)	0.022
Yes	58	5 (8.6)	1 (16.7)	0 (0.0)	0 (0.0)	4 (12.9)	0.585

*ATAAD* acute type A aortic dissection, *ICU* intensive care unit, *NA* not applicable.

The results of secondary outcomes were shown in the supplements (Supplemental Table 2). The results indicated that composite events (*P* for trend = 0.016), and elongated arrest time during surgery (at cutoff of 60 min, *P* for trend = 0.009; at cutoff of 90 min, *P* for trend = 0.005) exhibited a significant decreasing trend as the surgeon operative volume increased. The 4 groups did not differ significantly in terms of bypass time, clamp time, length of ICU stay, or length of hospital stay. We also considered different types of surgical procedures in this study. For procedures involving any extension from the ascending aorta, an elongated arrest time (*P* for trend = 0.001) exhibited a significant decreasing trend as the surgeon operative volume increased. For procedures that involved the ascending aorta only, composite events (*P* for trend = 0.029) and elongated clamp time (*P* for trend = 0.005) tended to be observed in the group with less operative volume.

**Figure 2:**
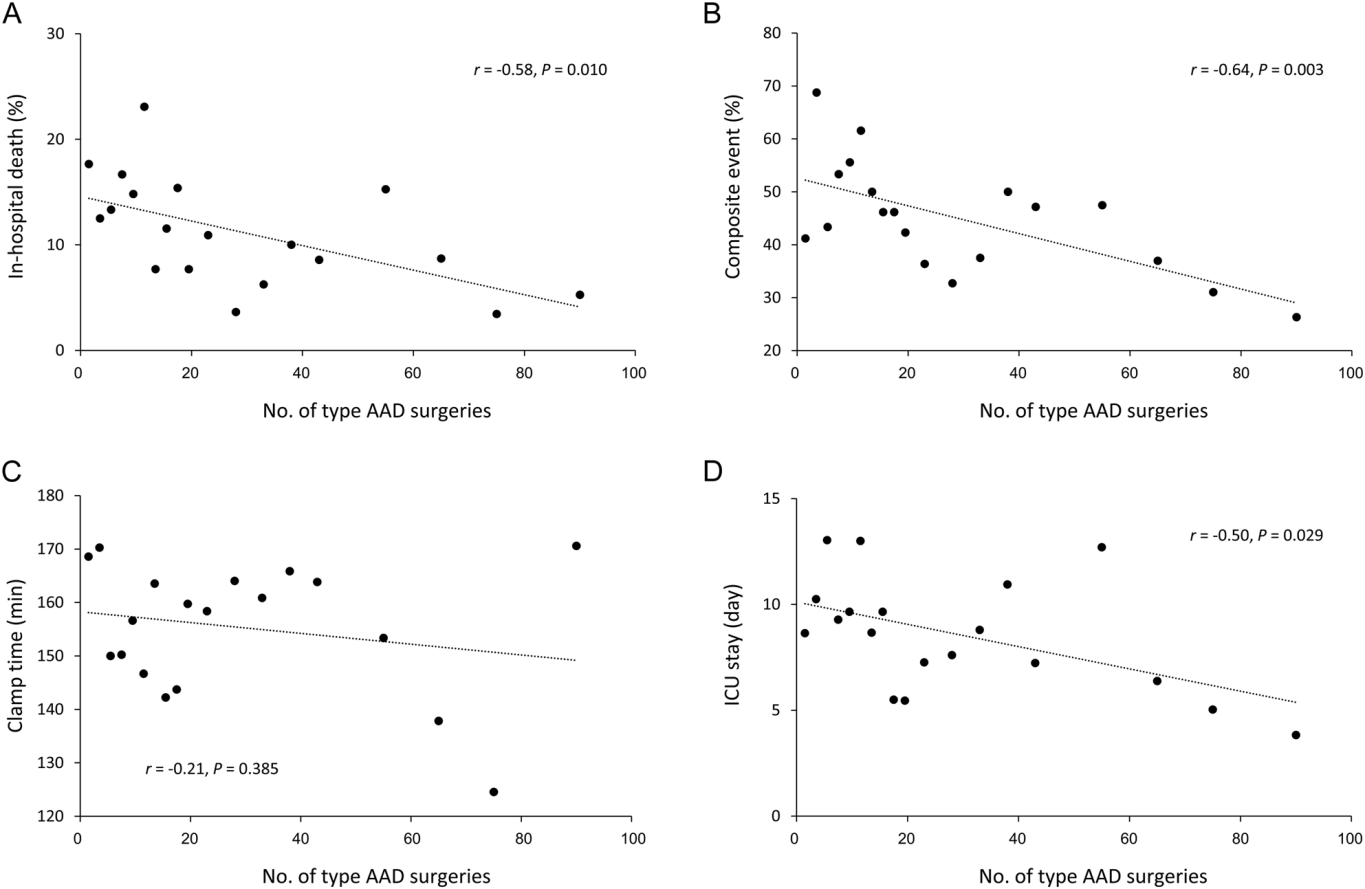
The relationship between the surgeon experience volume (defined as the number of AAD surgery of the surgeon) and the in-hospital mortality rate (**A**), the proportion of composite event (**B**), clamp time (**C**), and length of ICU stay (**D**). Composite event is anyone of in-hospital mortality, new-onset stroke, new-onset dialysis, respiratory failure, use of extracorporeal membrane oxygenation, and massive blood transfusion (packed red blood cell > 10U). Each point consists of 2 sequential numbers of operation volume among the 1st to 19th operations of the surgeon, 5 sequential numbers of operation volume among the 20th to 39th operations of the surgeon, and 10 sequential numbers of operation volume after the 40th operations of the surgeon, respectively. ICU, intensive care unit.

Additionally, we treated surgeon operative volume as a continuous variable. Accordingly, a higher surgeon operative volume was significantly correlated with a lower in-hospital mortality rate (*r* =  − 0.58, *P* = 0.010; Fig. 2A), lower risk of composite events (*r* =  − 0.64, *P* = 0.003; Fig. 2B), and shorter length of ICU stay (*r* =  − 0.50, *P* = 0.029; Fig. 2D). However, surgeon operative volume did not exhibit a significant correlation with clamp time (*r* =  − 0.21, *P* = 0.385; Fig. 2C).

**Figure 3:**
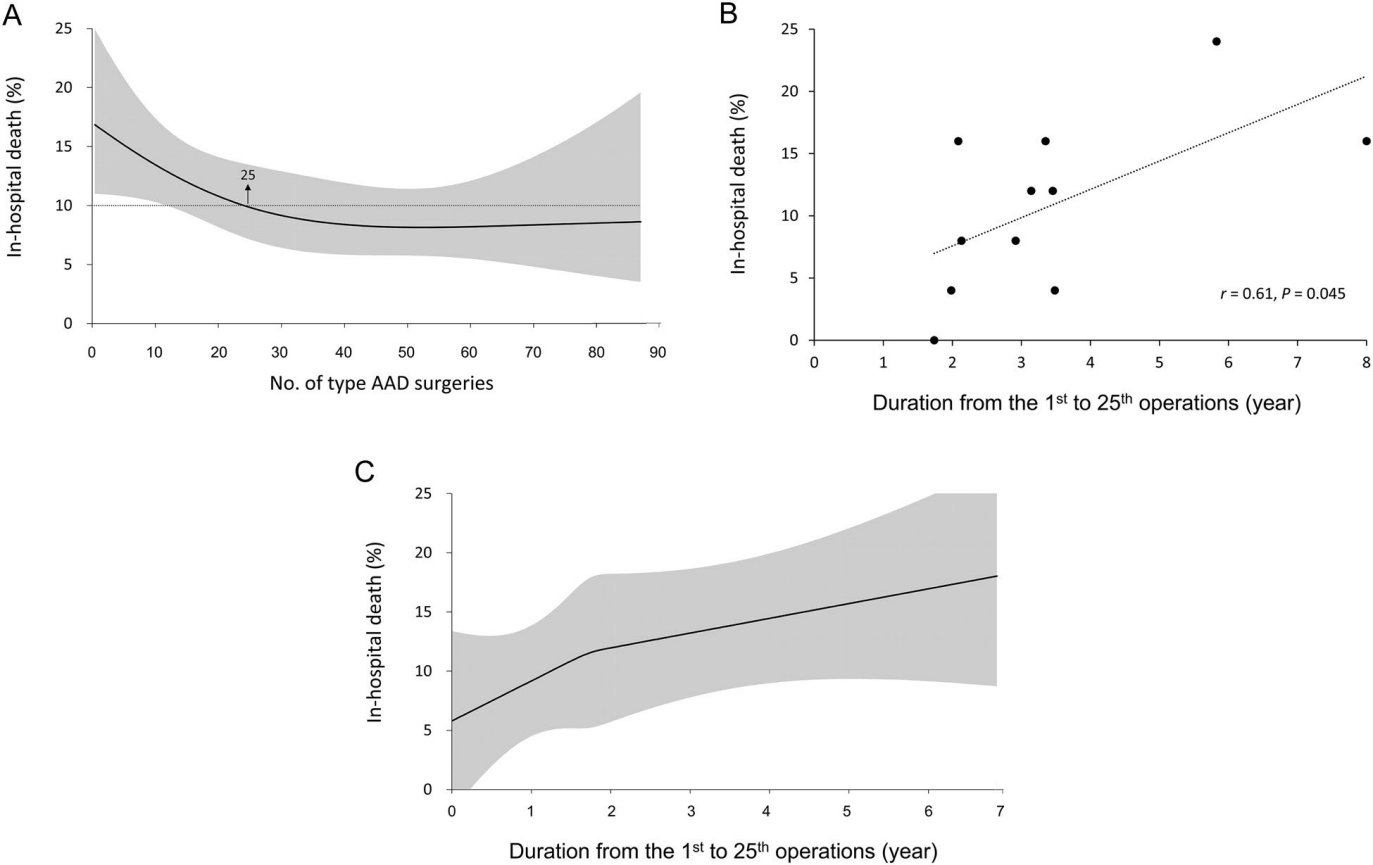
The non-linear relationship between the surgeon experience volume (defined as the sequential number of open surgical procedures for ATAAD performed by the surgeon after January 1, 2005) and in-hospital mortality rate (**A**). The relationship (**B**) and non-linear relationship (**C**) between the duration from the 1st to 25th operations of the surgeon and in-hospital mortality rate. ATAAD, acute type A aortic dissection.

We also defined a satisfactory mortality rate for operations performed by a surgeon as an average in-hospital patient mortality rate of 10%. The RCS model revealed that the optimal volume of operations to be performed by a surgeon was 25. For a surgeon with an experience operative volume above 25 for ATAAD, the average in-hospital mortality rate of the patients could be < 10% (Fig. 3A). Furthermore, the results showed that a longer duration from the 1st operation to the 25th operation by a surgeon was significantly correlated with a higher average in-hospital mortality rate of the patients (*r* = 0.61, *P* = 0.045; Fig. 3B). The relationship between the duration to the optimal operative volume and average in-hospital mortality rate was also linear in general (Fig. 3C).

## Discussion

In this study, we examined the relationship between surgeon operative volume and the corresponding outcomes of surgical repair of ATAAD. We obtained 3 prominent findings. First, a prominent learning curve exists for surgical repair of ATAAD: experience accumulation was associated with improved patient outcomes and surgical safety. Second, a surgeon operative volume of > 25 was related to an optimal in-hospital mortality rate, which was defined as ≤ 10%. Third, the duration to the optimal surgeon operative volume (25 operations) considerably influenced the patient outcomes, as a shorter duration was independently associated with more favorable surgical outcomes.

In general, ATAAD is a lethal cardiovascular disease^18^, with its reported in-hospital mortality rate ranging from 25 to 13%^8,9,11,14,15^. Due to its emergent and unpredictable nature^19^, numerous patients receive surgery performed by an on-duty surgeon under an emergent situation rather than under a well-scheduled setting. The chance of such surgery being performed by a junior surgeon, responsible for most duty work, is high. Hence, identifying the nature of the learning curve and implementing a relatively efficient training policy are crucial, This is particularly necessitated by the trend of a high proportion of patients receiving surgical repair rather than medication management in recent year^9,20^. Furthermore, surgery for ATAAD is a time-consuming process and considerably requires sustained attention, this thus renders such surgery a challenging task for aging surgeons. Accordingly, before surgeons are allowed to independently perform surgery for ATAAD, they must be provided with the relevant training that can enable them to achieve ideal perioperative outcomes.

Regarding surgery for ATAAD, studies have explored the relationship between either surgeon cumulative operative volume or hospital volume and patient outcomes and have reported nearly identical results^11,14–16^. A larger operative volume is associated with more favorable patient outcomes. This finding is consistent with that of our analysis of cumulative operative volume per surgeon (Fig. 1B). However, current evidence cannot explicitly identify the learning curve of a junior surgeon because related studies are limited to the annual hospital volume, cumulative hospital volume, cumulative surgeon volume, or annual surgeon volume. Due to incapability of defining each surgeon’s sequential volume of surgery, outcome analysis results could be obscured by the lack of data regarding the chronological relationship among surgical procedures for each surgeon. A surgeon’s initial surgical performance may bias their subsequent surgical performance when conducting outcome analysis. In such situations, the optimal operative volume required for a surgeon to achieve optimal ATAAD treatment outcomes could not be determined. We used data on the sequential operative volume of surgeons, including their every index operations, to analyze the relationship between operative volume and patient outcomes.

We analyzed 704 patients with a first-time diagnosis of ATAAD who received surgery; the results revealed a relatively low mortality rate for these patients (10.8%). This low mortality rate reflects that most patients with ATAAD in the CGRD were operated in the tertiary center. More ATAAD cases would be encountered in such aortic center in certain time period. Additionally, this finding can be attributed to the advanced recognition, increased clinical suspicion, and improved management of ATAAD^21^. Our results also reveal a significant learning curve for surgical repair of ATAAD. The relationship of in-hospital mortality rate, composite event rate, and length of ICU stay with surgeon operative volume exhibited a significant sliding trend. This demonstrates that increased operative volume is associated with improved outcomes and surgery related parameters.

When analyzing the impact of preoperative clinical condition and surgical procedure on in-hospital mortality, we found that the high-volume surgeon group would achieve better outcome in more severe clinical condition, such as pre-operative tamponade or shock, longer duration from onset to operation room, and deBakey I aortic dissection. More complicated surgical procedure, for those with surgeries involving any surgical extension, demonstrated similar relationships.

We defined an ideal mortality rate for surgical repair of ATAAD as an in-hospital patient mortality rate of < 10%. We found that surgeons with a cumulative operative volume of > 25 could achieve this ideal rate, on average. This can thus be set as the target volume for junior surgeons, which can enable them to achieve optimal outcomes and operate independently without a supervisor. However, in addition to operative volume, frequency plays a major role in achieving favorable outcomes. After comparing the training period of junior surgeons achieving 25 operations during the study period, we observed that a long training period can eliminate the positive effect of a large operative volume (Fig. 3). Hence, this finding confirms that fostering high-volume surgeons at high-volume hospitals can achieve the best outcome in terms of in-hospital mortality prevention. This is because high-volume hospitals such as tertiary referral centers encounter frequent ATAAD cases. In addition, high-volume supervisors at high-volume hospitals can guarantee better outcomes even if surgery is performed by low-volume surgeons^22^. Therefore, setting an adequate cooperation policy for junior surgeons is crucial to ensure improved surgical outcomes, as is establishing an aortic center or aortic subspecialty within a hospital to specialize in aortic surgery. This can not only facilitate the rapid accumulation of relevant experiences but also provide a robust backup and supervision system for patient surgery.

### Limitation

The study has multiple limitations. First, we could not systematically analyze the assistance involved in the operation, because no related detail, such as the experience or the assisting time of each supervisor during the operation, was claimable in the database. There might be the supervisor or trainee joined during the operation. However, the trainees in the Chang Gung Memorial Hospital systems had been limited for research fellows majoring in cardiovascular surgery. We consider the influence of the supervisor would gradually diminish as the operator’s volume increased, which would not be the significant cause for the improving outcome related to increased volume demonstrated in the study. Second, although the CGRD is the largest hospital-based database in Taiwan, our result should not be directly generalized to other medical institutions. Considering different hospital scales, the optimal operative volume of 25 suggested in this study might differ for other hospitals. Third, there might be generation effect in surgical outcome due to the evolution in perioperative care and surgical technique of ATAAD in recent years. The junior surgeons trained in recent years might achieve a better outcome in their early career surgeries compared to those trained in older years because standing on the shoulder of the giant. Finally, because of our study’s retrospective design, we could infer only associations rather than causation.

## Conclusion

Surgery for ATAAD involves a prominent learning curve in terms of improving clinical outcomes and achieving surgical safety. We observed that surgeons with a cumulative operative volume of > 25 could achieve the goal of an in-hospital patient mortality rate of < 10%; however, a relatively long training period might eliminate the positive effect engendered by a large operative volume. Hence, we suggest that fostering high-volume surgeons at high-volume hospitals and transferring patients to high-volume hospital can achieve the best outcome regarding the prevention of in-hospital mortality.

## Supplementary Information

Supplementary Tables.

## Data Availability

The datasets generated and analyzed during the current study are not publicly available due to the policy and regulation of the Institutional Review Board of Chang Gung Memorial Hospital, but are available from the corresponding author on reasonable request.
