# Supplementary material for: Learning curve for open surgical repair of acute type A aortic dissection
Source: Sci Rep. 2023 Mar 3;13:3601. doi: 10.1038/s41598-023-30397-2 (PMC9984377; doi:10.1038/s41598-023-30397-2)
Supplement: Supplementary file 1 — Supplementary Tables. [file 41598_2023_30397_MOESM1_ESM.docx]

**SUPPLEMENT MATERIAL**

**Supplemental Table 1.** The in-hospital mortality and composite event of each surgeon

| Number of surgeon | Total volume between 2005 and 2018 | In-hospital mortality,  n (%) | Composite event,  n (%)* |
| --- | --- | --- | --- |
| 1 | 97 | 3 (3.1) | 32 (33.0) |
| 2 | 82 | 8 (9.8) | 31 (37.8) |
| 3 | 76 | 4 (5.3) | 30 (39.5) |
| 4 | 73 | 5 (6.8) | 26 (35.6) |
| 5 | 66 | 12 (18.2) | 33 (50.0) |
| 6 | 59 | 7 (11.9) | 35 (59.3) |
| 7 | 50 | 7 (14.0) | 24 (48.0) |
| 8 | 40 | 5 (12.5) | 19 (47.5) |
| 9 | 35 | 6 (17.1) | 17 (48.6) |
| 10 | 33 | 3 (9.1) | 14 (42.4) |
| 11 | 30 | 0 (0.0) | 7 (23.3) |
| 12 | 20 | 5 (25.0) | 14 (70.0) |
| 13 | 20 | 4 (20.0) | 15 (75.0) |
| 14 | 9 | 1 (11.1) | 5 (55.6) |
| 15 | 8 | 6 (75.0) | 8 (100.0) |
| 16 | 4 | 0 (0.0) | 2 (50.0) |
| 17 | 2 | 0 (0.0) | 0 (0.0) |

* Anyone of in-hospital mortality, new-onset stroke, new-onset dialysis, respiratory failure, use of extracorporeal membrane oxygenation and massive blood transfusion (packed red blood cell >10U).

**Supplemental Table 2.** Perioperative outcomes of the patients according to the cumulative operation volume of the surgeon

|  |  | Number of ATAAD surgery of the surgeon | | | |  |
| --- | --- | --- | --- | --- | --- | --- |
| Subgroup / Surgical extension | Total | ≤10th | 11th to 20th | 21st to 30th | >30th | *P* trend |
| Total |  |  |  |  |  |  |
| Number | 704 | 153 | 130 | 110 | 311 |  |
| In-hospital death | 76 (10.8) | 23 (15.0) | 17 (13.1) | 8 (7.3) | 28 (9.0) | 0.031 |
| Composite event ^#^ | 312 (44.3) | 80 (52.3) | 64 (49.2) | 38 (34.6) | 130 (41.8) | 0.016 |
| Bypass time, min | 249.7 ± 79.7 | 249.8 ± 70.9 | 238.3 ± 79.4 | 263.1 ± 85.0 | 249.4 ± 81.1 | 0.670 |
| Bypass time >240 min | 248 (49.8) | 53 (57.0) | 40 (46.0) | 44 (57.1) | 111 (46.1) | 0.149 |
| Bypass time >180 min | 422 (84.7) | 80 (86.0) | 68 (78.2) | 69 (89.6) | 205 (85.1) | 0.665 |
| Clamp time, min | 155.6 ± 53.2 | 159.5 ± 48.7 | 151.1 ± 55.0 | 161.2 ± 48.2 | 154.0 ± 55.7 | 0.603 |
| Clamp time >180 min | 135 (26.5) | 29 (31.2) | 24 (27.3) | 26 (32.9) | 56 (22.5) | 0.099 |
| Clamp time >120 min | 371 (72.9) | 74 (79.6) | 58 (65.9) | 62 (78.5) | 177 (71.1) | 0.362 |
| Arrest time, min | 45.3 ± 21.8 | 47.9 ± 23.6 | 43.5 ± 20.7 | 42.9 ± 17.3 | 45.6 ± 22.8 | 0.622 |
| Arrest time ≥90 min | 8 (2.1) | 5 (6.2) | 2 (2.7) | 0 (0.0) | 1 (0.6) | 0.005 |
| Arrest time ≥60 min | 67 (17.9) | 23 (28.4) | 13 (17.8) | 8 (14.3) | 23 (13.9) | 0.009 |
| ICU stay, day | 4 [2, 8] | 5 [2, 9] | 4 [2, 8] | 4 [3, 8] | 4 [2, 9] | 0.314 |
| ICU stay >7 day | 203 (29.0) | 45 (29.6) | 37 (28.7) | 30 (27.3) | 91 (29.4) | 0.979 |
| Hospital stay, day | 18 [12, 28] | 18 [12, 31] | 17 [12, 25] | 18 [12, 27] | 17 [12, 28] | 0.653 |
| Hospital stay >30 day | 156 (22.2) | 39 (25.5) | 24 (18.5) | 25 (22.7) | 68 (21.9) | 0.637 |
| Any extension |  |  |  |  |  |  |
| Number | 287 | 60 | 53 | 52 | 122 |  |
| In-hospital death | 39 (13.6) | 11 (18.3) | 10 (18.9) | 5 (9.6) | 13 (10.7) | 0.080 |
| Composite event ^#^ | 137 (47.7) | 30 (50.0) | 31 (58.5) | 21 (40.4) | 55 (45.1) | 0.247 |
| Bypass time, min | 282.4 ± 85.0 | 270.5 ± 83.9 | 297.9 ± 76.5 | 292.2 ± 102.9 | 276.9 ± 81.2 | 0.770 |
| Bypass time >240 min | 128 (67.7) | 20 (71.4) | 24 (72.7) | 25 (73.5) | 59 (62.8) | 0.246 |
| Clamp time, min | 183.9 ± 55.1 | 173.3 ± 55.0 | 193.4 ± 49.8 | 186.0 ± 53.3 | 182.8 ± 57.7 | 0.859 |
| Clamp time >180 min | 95 (48.5) | 12 (42.9) | 21 (60.0) | 19 (54.3) | 43 (43.9) | 0.490 |
| Arrest time, min | 54.5 ± 23.2 | 59.4 ± 29.2 | 62.4 ± 19.8 | 48.9 ± 20.5 | 52.5 ± 22.3 | 0.072 |
| Arrest time ≥90 | 8 (5.4) | 5 (20.0) | 2 (8.3) | 0 (0.0) | 1 (1.4) | 0.001 |
| ICU stay, day | 4 [2, 9] | 5 [3, 10] | 5 [2, 10] | 4 [3, 8] | 4 [2, 9] | 0.999 |
| ICU stay >7 day | 83 (29.1) | 19 (31.7) | 18 (34.6) | 14 (26.9) | 32 (26.5) | 0.316 |
| Hospital stay, day | 16 [12, 27] | 18 [12, 28] | 16 [12, 25] | 15 [11, 26] | 17 [12, 28] | 0.604 |
| Hospital stay >30 day | 59 (20.6) | 12 (20.0) | 10 (18.9) | 10 (19.2) | 27 (22.1) | 0.669 |
| Ascending only |  |  |  |  |  |  |
| Number | 417 | 93 | 77 | 58 | 189 |  |
| In-hospital death | 37 (8.9) | 12 (12.9) | 7 (9.1) | 3 (5.2) | 15 (7.9) | 0.184 |
| Composite event ^*^ | 175 (42.0) | 50 (53.8) | 33 (42.9) | 17 (29.3) | 75 (39.7) | 0.029 |
| Bypass time, min | 229.3 ± 68.9 | 240.9 ± 63.1 | 200.8 ± 54.8 | 239.4 ± 58.2 | 231.7 ± 76.1 | 0.797 |
| Bypass time >240 min | 120 (38.8) | 33 (50.8) | 16 (29.6) | 19 (44.2) | 52 (35.4) | 0.133 |
| Clamp time, min | 137.8 ± 43.5 | 153.5 ± 44.8 | 123.2 ± 37.9 | 141.5 ± 32.8 | 135.2 ± 45.5 | 0.074 |
| Clamp time >180 min | 40 (12.8) | 17 (26.2) | 3 (5.7) | 7 (15.9) | 13 (8.6) | 0.005 |
| Arrest time, min | 39.3 ± 18.6 | 42.7 ± 18.7 | 34.6 ± 14.1 | 37.2 ± 11.3 | 40.3 ± 21.8 | 0.853 |
| Arrest time ≥90 | 0 (0.0) | 0 (0.0) | 0 (0.0) | 0 (0.0) | 0 (0.0) | NA |
| ICU stay, day | 4 [2, 8] | 5 [2, 8] | 4 [2, 7] | 4 [2, 8] | 4 [2, 9] | 0.093 |
| ICU stay >7 day | 120 (28.9) | 26 (28.3) | 19 (24.7) | 16 (27.6) | 59 (31.2) | 0.438 |
| Hospital stay, day | 18 [12, 28] | 18 [13, 33] | 18 [11, 25] | 20 [13, 34] | 18 [12, 27] | 0.066 |
| Hospital stay >30 day | 97 (23.3) | 27 (29.0) | 14 (18.2) | 15 (25.9) | 41 (21.7) | 0.354 |

ATAAD, Acute type A aortic dissection; ICU, intensive care unit; NA, not applicable;

* Anyone of in-hospital mortality, new-onset stroke, new-onset dialysis, respiratory failure, use of extracorporeal membrane oxygenation, and massive blood transfusion (packed red blood cell >10U).
